# FedAvg-P: Performance-Based Hierarchical Federated Learning-Based Anomaly Detection System Aggregation Strategy for Advanced Metering Infrastructure

**DOI:** 10.3390/s24175492

**Published:** 2024-08-24

**Authors:** Hend Alshede, Kamal Jambi, Laila Nassef, Nahed Alowidi, Etimad Fadel

**Affiliations:** 1 Department of Computer Science, Faculty of Computing and Information Technology, King Abdulaziz University, Jeddah 21589, Saudi Arabia; kjambi@kau.edu.sa (K.J.); lmohamed@kau.edu.sa (L.N.); nalowidi@kau.edu.sa (N.A.); eafadel@kau.edu.sa (E.F.); 2 Self-Development Skills Department, Common First Year Deanship, King Saud University, Riyadh 12211, Saudi Arabia

**Keywords:** advanced metering infrastructure, CICIDS2017, anomaly detection system, hierarchical federated learning, aggregation strategy, SPoF, peer to peer

## Abstract

Advanced metering infrastructures (AMIs) aim to enhance the efficiency, reliability, and stability of electrical systems while offering advanced functionality. However, an AMI collects copious volumes of data and information, making the entire system sensitive and vulnerable to malicious attacks that may cause substantial damage, such as a deficit in national security, a disturbance of public order, or significant economic harm. As a result, it is critical to guarantee a steady and dependable supply of information and electricity. Furthermore, storing massive quantities of data in one central entity leads to compromised data privacy. As such, it is imperative to engineer decentralized, federated learning (FL) solutions. In this context, the performance of participating clients has a significant impact on global performance. Moreover, FL models have the potential for a Single Point of Failure (SPoF). These limitations contribute to system failure and performance degradation. This work aims to develop a performance-based hierarchical federated learning (HFL) anomaly detection system for an AMI through (1) developing a deep learning model that detects attacks against this critical infrastructure; (2) developing a novel aggregation strategy, FedAvg-P, to enhance global performance; and (3) proposing a peer-to-peer architecture guarding against a SPoF. The proposed system was employed in experiments on the CIC-IDS2017 dataset. The experimental results demonstrate that the proposed system can be used to develop a reliable anomaly detection system for AMI networks.

## 1. Introduction

An advanced metering infrastructure (AMI) is the backbone of a smart grid, which establishes two-way communication between each consumer and the provider’s control center. The AMI allows consumers to access historical data regarding energy consumption and facilitates dynamic pricing, which may reduce the peak load. At the same time, utility companies are served by being provided with a more detailed picture of consumer energy consumption, allowing them to enact plans to reduce some of their financial burdens [1].

An AMI is essentially a network comprising three interconnected types of components, namely (1) smart meters (SMs), (2) data concentrators (DCs), and (3) headend systems, all of which are tied together through a heterogeneous combination of communications channels and protocols [2]. In terms of network topology, AMI networks typically present a hierarchical-based tree topology with little or no redundancy due to scalability concerns and deployment cost constraints. The headend serves as the hub of several data concentrators, where a data concentrator is the data collector for metering data from numerous smart meters [3]. The infrastructure is constrained by a three-tier network architecture. A wide area network (WAN) connects data concentrators to the headend system, while a neighbourhood area network (NAN) connects these data concentrators to smart meters. A home area network (HAN) connects a particular customer’s smart meter to their home appliances [4]. First, HANs aggregate massive metering data from smart meters in various households. A data concentrator receives energy usage data and metering data from multiple data concentrator points within the NAN. A headend then deeply analyzes this high-volume collected data, providing a summary of individual behavior patterns and power utilization to enable precise real-time control and efficient resource allocation [5].

Moreover, each device within an AMI can be considered a discrete object containing a unique Internet Protocol (IP) address, allowing it to upload data and download instructions through the Internet [6]. This infrastructure makes an AMI highly prone to cyberattacks such as energy fraud and theft, data theft, deliberate service interruptions aimed at extortion, sabotage, terrorism, hacktivism, and innumerable other threats. The large-scale implementation of an AMI heightens the risk of an attack, posing a hazardous problem and generating significant economic losses. Such losses are not only financial but may entail a genuine threat to the lives and safety of consumers [7]. The zero-day attack (ZDA) [8] is one of the most severe threats, exploiting previously unknown network vulnerabilities that are not included in training models. With their high success rate, zero-day attacks typically target high-value targets such as critical infrastructure and financial and medical institutions [9]. Therefore, attackers keep newly discovered vulnerabilities confidential for as long as possible, maximizing the possibility of an attack succeeding [10]. Hence, reliably defending against such attacks requires exploring alternative solutions due to their nature, which has not been seen before [11].

Federated learning (FL) is a decentralized method of machine learning (ML) that allows cohorts of users to collectively train a shared model through the exchange of model parameters rather than relying on streams of raw data sent to cloud servers, which are inherently vulnerable to attack [12]. In federated learning, clients compute updates for the server, and they only communicate this update. These updates are specifically deployed to enhance current ML models and do not require storage once applied, ensuring data privacy. Federated learning also shields the cloud from potential attacks, restricting the attack surface to only the device. The added benefits of reduced latency and network bandwidth requirements make federated learning attractive for AMI services [13]. Conceptually, federated learning revolves around two primary components: data clients and an aggregation server [14]. It is generally iterated in three steps as follows: first, the aggregation server stores a global model and sends it to its clients; second, the data clients utilize their local training data and communicate the shared local training parameters with the aggregation server; and finally, the aggregation server updates its global model based on the received shared parameters by incorporating the shared parameters according to an aggregation strategy. The FL method has benefits in terms of the use of low-cost ML models on devices such as sensors and cell phones. In addition, FL permits algorithms to acquire experience, which is another feature that conventional machine learning techniques do not always guarantee [15].

Traditional federated learning frameworks offer many advantages, but many problems remain unresolved. Some of these limitations affect the system’s performance. In this work, we are concerned with enhancing the performance of the anomaly detection system due to the critical nature of AMIs and the substantial damage when disrupted. Thus, we summarize the significant limitations as follows: (1) System heterogeneity—in federated learning, the local performance of devices may differ; therefore, selecting lazy clients will likely impede the training process. (2) Single Point of Failure (SPoF)—the resilience of an aggregator is contingent on the robustness of the central server, and, if the aggregator fails, the entire FL network will collapse. (3) Malicious clients and false data—poisoning attacks that contaminate the updated parameters can seriously harm the global model’s performance [16].

In this context, this work aims to develop a performance-based Hierarchical Federated Learning-based Anomaly Detection System (HFL-ADS) to detect attacks targeting the communication infrastructure of AMIs. The contributions to this work are as follows: (1) This work investigates different deep learning algorithms to find the most effective one for detecting known and zero-day attacks; then, we develop HFL-ADS for the AMI network, which can detect most attacks against it. (2) This work develops a novel aggregation strategy, FedAvg-P, to enhance the global performance of HFL-ADS systems. FedAvg-P relies on a client selection (CS) mechanism based on the client’s model performance to use within the FL system. In addition, it identifies malicious smart meters in FL to protect the training process against a malicious participant. (3) This work proposes a peer-to-peer HFL-ADS architecture that can mitigate the SPoF issue in traditional HFL and create a secure and reliable system. (4) We validated, compared, and simulated the results of the proposed system. First, we validated the proposed deep learning system by comparing it with state-of-the-art deep learning models; then, we validated the proposed client selection mechanism by comparing its results to a random selection strategy; next, we validated the proposed system through simulated poisoning attacks; and last, we performed a comprehensive simulation of the proposed system with the proposed mechanisms.

## 2. Literature Review

**Figure 1:**
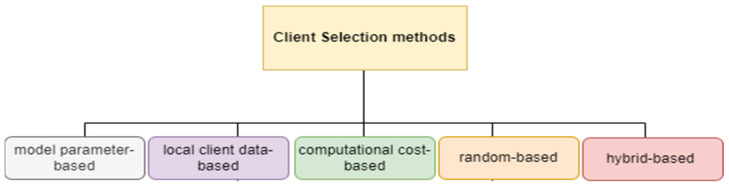
Grouping of CS methods used in FL systems.

Typical applications of FL are marked by significant heterogeneity in client performance. As such, randomly selecting clients for each training cycle does not completely exploit the clients’ heterogeneous local updates, thereby diminishing the model’s accuracy, engendering a slower convergence rate, degrading fairness, and having other harmful effects. Several client selection algorithms that promise improved performance have been developed to tackle the problem of FL client heterogeneity [17]. These can be classified into five groups, namely model parameter-based, local client data-based, computational resources-based, random-based, and hybrid-based client selection strategies, as shown in Figure 1.

Model parameter-based methods assess a number of chosen local parameters that have the potential to impact the global model and assign a higher probability of selection to higher-performing clients. In contrast, local client data-based methods divide clients into clusters according to the similarity of their local data distribution, allowing for federated optimization within each cluster to significantly enhance the model’s performance and efficiency [18]. The computational resource-based methods primarily involve clients sending their resource information to the server, including communication status, computing resource size, and other critical data. The server then utilizes these data to estimate the time required for updates and determine which clients to select for training [19]. The primary FL client selection method is random selection, which merely selects clients randomly [20]. Finally, hybrid-based client selection methods combine two or more of the abovementioned methods.

**Figure 2:**
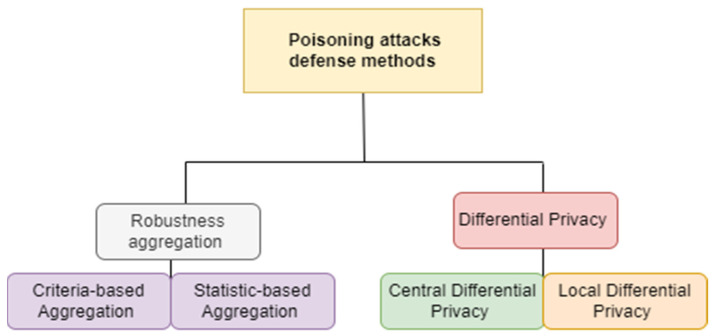
Defense methods against poisoning attacks.

In a poisoning attack, the attacker tries to manipulate the global FL model to degrade its performance. Two types of poisoning attacks can result in poisoned updates: (1) a data poisoning attack while gathering local data; and (2) a model poisoning attack, which disrupts the local model training process. Both types of poisoning attacks aim to modify a target model’s behavior in an undesirable way. The defense methods against poisoning attacks are robustness aggregation and Differential Privacy (DP) [21,22] (Figure 2).

In the robustness aggregation defense method, the aggregation server can check the local model’s performance using a validation dataset. The aggregation server continuously assesses whether the updates from hacked clients differ statistically from those received from unhacked clients. This method comprises two categories: criteria-based methods, which adjust the weights of local models according to certain criteria, and statistics-based methods, which utilize statistical approaches to design aggregation algorithms.

DP provides a way of protecting against threats by adding a small amount of noise with a tiny standard deviation to the global model. This keeps attackers from obtaining private information from the training dataset [23]. DP applications may be further divided into two categories [24]: (1) Central Differential Privacy (CDP), a centralized means of protection in which the user trusts the central database holder; and (2) Local Differential Privacy (LDP), which overcomes the inherent problem of trust in CDP through having individuals encode or modify their responses with random noise before submitting them to the central server.

Sarhan et al. [25] proposed an ML attack detection system in which HFL is used to protect system data and the learning process. The architecture was developed by combining HFL and blockchain concepts. Moreover, a smart contract was used to achieve conformance with executed tasks based on specific criteria. They used a deep feed-forward (DFF) algorithm to train and validate the framework. They used the NF-BoT-IoT-v2 dataset to build their model. The results showed reliable DoS detection, with an accuracy of 95.77%, whereas an accuracy of only 63.88% was obtained for theft detection.

Sun et al. [26] presented a transformer-based Intrusion Detection System (Transformer-IDM), which aims to enhance the intrusion detection rate. They integrated 5G technology into the AMI network. Transformer-IDM is based on a hierarchical federated learning IDS to protect consumer privacy in the AMI system. They used the NSL-KDD dataset to build the model. The results showed that the suggested intrusion detection obtained high results (of about 99%) for accuracy, precision, F1 score, and recall.

Schueller et al. [27] utilized the architecture of a hierarchical ADS for software-defined networks (SDNs), which integrates the benefits of a flow-based ADS and a packet-based ADS to increase the attack detection rate (DR) without degrading the SDN performance. The proposed ADS is based on a support vector machine (SVM). The results on the DARPA dataset demonstrated a high packet-based DR of 99.42% and an error rate (ER) of 12%.

Rashid et al. [28] proposed an FL-based IDS for Industrial Internet of Things (IIoT) networks. The proposed model ensures that IoT devices process data privately and securely. They used a new dataset, named the Edge-IIoT dataset to evaluate the proposed method. The results indicated that the suggested FL model achieved an accuracy of 92.49%.

Additionally, Wang et al. [29] proposed a particular IDS comprising federated transfer learning (FTL) and extreme learning machine (ELM) technologies, termed FLTrELM, which promises to enhance intrusion detection through the integration of federated data aggregation. First, FLTrELM constructs an ELM model to address the issues of inadequate samples and probability adaptation. After that, it utilizes the model to learn strategies for data privacy protection that do not require training data to be shared, ultimately producing an attack detection model. Based on tests carried out on the NSL-KDD, KDD99, and ISCX2012 datasets, the suggested model achieved higher DR results and robust performance even in the face of small samples and unprecedented intrusions, ensuring data privacy.

Ayache et al. [30] presented an enhancement of the existing Cognitive Internet of Medical Things (CIoMT) framework, which uses blockchain and FL technologies. The proposed model provided a safe and dependable distributed framework. They proposed blockchain-based FL for two reasons: first, the blockchain decentralized architecture resolves the problem of an SPoF. Second, the consensus mechanism of blockchain offers authentication tests that prevent malicious participants. The results indicate that the performance of the proposed method provides substantial benefits when compared to conventional methods.

Putra et al. [31] proposed an FL architecture for IIoT with a novel CS method to increase global model accuracy. The proposed CS, called ACS, is based on the accuracy of the local model. ACS selects the highest-performing clients to share the global model. The evaluation of ACS was performed using the MNIST and F-MNIST datasets. The results showed that ACS improved the global accuracy and F1 score by around 4.62%.

Zeng et al. [32] focused on client selection with federated learning. They proposed a novel aggregation strategy called FedChoice, which is based on the client model’s loss function to improve the global model. Local models with high loss were set with a higher likelihood of being chosen in the global aggregation. They conducted experiments to validate their proposed strategy on the CIFAR-10, CINIC-10, MNIST, EMNITS, and FEMNIST datasets. The results demonstrated a significant enhancement in the performance of FedChoice. Table 1 summarizes the literature on FL-based detection systems.

## 3. Model Formulation

This work aims to develop a performance-based federated learning-based anomaly detection system that can protect AMI networks from attacks. Typically, smart meters deployed across vast locations can train global anomaly detection collaboratively, employing an exchange of model parameters with a headend system. As such, there is no requirement to transmit the data collected by smart meters to a headend system for centralized processing, thus securing privacy. The trained model monitors attacks against smart meters within this distributed system, triggering an alarm to protect the AMI system.

**Figure 3:**
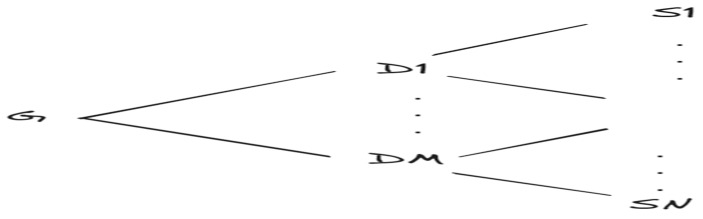
Representation of the system components.

Let G denote the global server, M the number of data concentrators as D = {*D_1_,…, D_M_*}, and N the number of smart meters as S = {*S_1_,…, S_N_*}, where M is less than N, as one data concentrator is connected to multiple smart meters. The system is represented in Figure 3:

The global server, G, sends the initial parameter $\omega_{i}^{\left(t\right)}$ to a random subset $\tilde{D\;}$ of data concentrators, where $\tilde{D\;}\in D$. Then, each data concentrator in $\tilde{D\;}$ sends $\omega_{i}^{\left(t\right)}\;$to all its connected smart meters in S. Then, each smart meter receives $\omega_{i}^{\left(t\right)}$ and trains a deep-learning model on its local data. This step aims to maximize the detection rate by testing different deep-learning models. For smart meter *S_i_* with local dataset $SD_{i}$ and $ɭ\left(\omega ;(x_{n},y_{n})\right)$ loss based on one data sample $\left(x_{n},y_{n}\right)$, the training process and total loss function are described in Equations (1) and (2), respectively [33]:

(1) $$\omega_{i}^{\left(t+1/2\right)}=\omega_{i}^{\left(t\right)}-\eta \nabla f_{i}\left(\omega_{i}^{\left(t\right)}\right)$$

(2) $$f_{i}(\omega )=\frac{1}{\left|{SD}_{i}\right|}\sum_{(x_{n},y_{n})\in \;{SD}_{i}}ɭ\left(\omega ;(x_{n},y_{n})\right)$$

Next, a smart meter *S_i_* can share its trained intrusion detection model collaboratively. Each trained smart meter will send three values to its data concentrator, namely the local model parameter $\omega_{i}^{\left(t+\frac{1}{2}\right)}$, local data size ${|SD}_{i}|$, and local F1 score $S_{i}$_F1_, as in Equation (3):

(3) $$\forall \;S_{i}\in D_{j}\;\mathrm{send}\text{\_}\mathrm{to}\_D_{j}\;(\omega_{i}^{\left(t+\frac{1}{2}\right)},\;{|SD}_{i}|,\;S_{iF1})$$

The goal in this step is to maximize the ability of the global server *G* to detect possible attacks, thereby maximizing the accuracy and F1 score. First, we propose a new client selection mechanism based on the local model parameters to choose high-performance clients. We aim to aggregate more updates by selecting the top-X clients with a higher F1 score in one round, as the F1 score offers a more reliable assessment metric, particularly in cases when the minority class is of considerable significance. In addition, the new client will be excluded from the system for model aggregation, which will degrade the global performance until it reaches a high training performance, as in Equation (4):

(4) $$\forall \;D_{j}\;\in \;\tilde{D\;},\;\mathrm{select}(TopX(S_{iF1}))$$

Second, we propose a criteria-based model poisoning attack defense model to detect and ban any malicious attack at this layer. We define a global testing set in each data concentrator where the top-X weight is tested, and we ban and delete smart meters that reach a pre-defined threshold *τ*.

However, based on these two techniques, we propose a novel secure aggregation method, FedAvg-Performance or FedAvg-P. It is described formulaically in Equation (5):

(5) $$\omega_{D_{j}}^{\left(t+1/2\right)}=\sum_{i\in D_{j}}^{topX}\left(\frac{\left|{SD}_{i}\right|}{\sum_{{i\in D}_{j}}\left|{SD}_{i}\right|}\omega_{i}^{\left(t+1/2\right)}\right)\;\mathrm{where}\;f_{i}\left(\omega_{i}^{\left(t+1/2\right)}\right)<\tau$$

where $\omega_{D_{j}}^{\left(t+1/2\right)}$ is the established NAN model for the data concentrator $D_{j}$.

Finally, we propose a peer-to-peer setting, where the averaging process also occurs on each data concentrator device $D_{j}$; this is in contrast to the traditional FL setup, where clients send their local model parameters and evaluation results to a central server that computes the average and returns the updated weights to the clients for the next learning round. The goal of this step is to create an architecture that is resistant to an SPoF. Each data concentrator $D_{j}$ sends its obtained weight to all other data concentrators in the set $\tilde{D\;},$ denoted as$\;D_{other}$. As a result, every data concentrator $D_{j}$ can obtain the weights from $D_{other}$. The proposed global aggregation is the classical aggregation method, FedAvg, which is described formulaically in Equation (6):

(6) $$\omega^{\left(t+1\right)}=\sum_{j\in \tilde{D}}\left(\frac{\sum_{{i\in D}_{j}}\left|{SD}_{i}\right|}{\left|SD\right|}\omega_{D_{j}}^{\left(t+1/2\right)}\right)$$

where $\omega^{\left(t+1\right)}\;$is the obtained global model and $\left|SD\right|=\sum_{j\in \tilde{D}}\left|{SD}_{j}\right|$ is the total size of all local datasets. This peer-to-peer communication approach offers several advantages over centralized federated learning. First, it reduces the possibility of data leaks. Furthermore, it enhances the system’s reliability by eliminating an SPoF. Additionally, it allows for more effective communication between nodes.

## 4. The Proposed HFL-ADS System

### 4.1. Proposed Deep Learning Model

**Figure 4:**
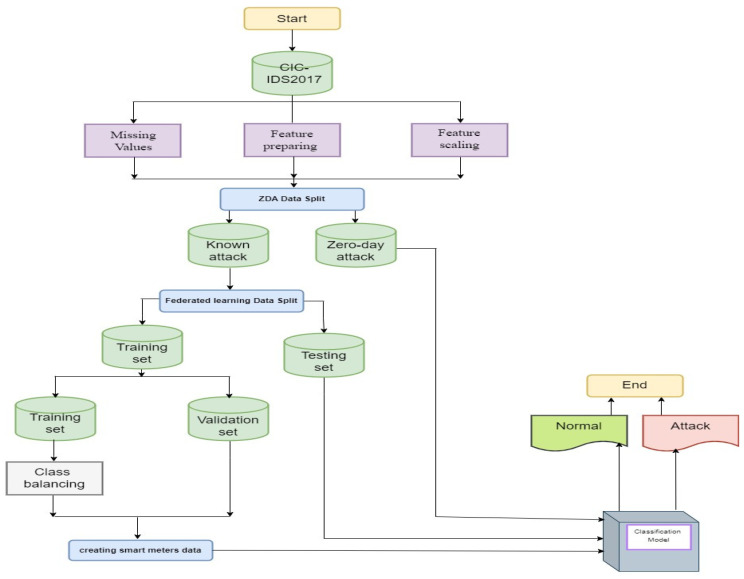
The proposed deep learning model.

The development of the deep learning model comprises three main stages: data collection, data pre-processing, and the development of a classification model. Figure 4 illustrates the proposed deep learning model.

#### 4.1.1. Data Collection

AMI networks are IP-based systems, and the same cyberattacks that afflict IT systems threaten to disrupt AMI systems [34]. In this work, the CICIDS2017 [35] dataset was used to build and validate the proposed model. CIC-IDS2017 is provided by the Canadian Institute of Cybersecurity (CIC) and is considered one of the most critical datasets as it contains up-to-date threats that are not represented by older datasets [36]. It includes 2,522,362 various flows, of which 2,096,484 are normal flows and 425,878 are attack flows with 79 various features. This dataset covers 15 different classes: A normal type and 14 attack types, including DoS Hulk, DDoS, PortScan, DoS GoldenEye, FTP Patator, DoS Slowloris, DoS Slowhttptest, SSH Patator, Bot, Web Attack: Brute force, Web attack: XSS, Infiltration, Web Attack: SQL injection, and Heartbleed.

ZDA Split: ZDA is conducted to predict classes not included in the training set and is primarily applicable when the training data provide an incomplete representation of all class categories of interest. Considering the growing prominence of zero-day attacks, this scenario is most feasible and relevant for ADS. Thus, the training and test instance classes are different: training instances are termed “seen classes”, whereas test instances are termed “unseen classes”. In the case of attack detection, seen classes represent those attacks that are known to the training model, whereas unseen classes are zero-day attacks. The original data, D, were split into two distinct groups of known and zero-day classes; in particular $D_{k}$ is the known dataset, while $D_{z}$ is the zero-day class, and they are described using the set notation in Equations (7) and (8):

(7) $$D_{k\;}\cap \;D_{z\;}=\emptyset$$

(8) $$D_{k\;}\cup \;D_{z\;}=D$$

Models are trained on $D_{k}$ instances and tested on $D_{k}$ and $D_{z}$ instances to obtain accuracy.

Federated learning data split: the primary concern of federated learning is to create models through distributed datasets that protect against data leakage. To build the global model, the $D_{k\;}\;$dataset for known attacks is divided into a training set ${TrD}_{k\;}$ and a testing set $TeD_{k}$. The set ${TrD}_{k}$ is divided between smart meters to train and validate local models. For each class (normal or attack), we assign a random percentage *C* for each smart meter $S_{i}$, such that the sum of all percentages for each class is 1, as in Equations (9) and (10):

(9) $$\forall \;S_{i}\in S_{n}\;,\;\mathrm{assign}C_{iN\;}\;C_{iA\;}\;,\;\sum_{1}^{n}C_{iN\;,\;}=1\;and\;\sum_{1}^{n}C_{iA\;,\;}=1$$

(10) $${SD}_{i}\;\leftarrow \;{TrD}_{k\;}\left[normal\right]\left(\left|normal\right|*C_{iN\;,\;}\right)+{TrD}_{k\;}\left[attack\right]\left(\left|attack\right|*C_{iA\;,\;}\right)$$

where $SD_{i}$ is the local dataset for the smart meter $S_{i}$, $C_{iN\;}$ is the percentage of normal instances for meter $S_{i}$, $C_{iA\;}$ is the percentage of attack instances for smart meter $S_{i}$, $\left|normal\right|$ is the number of normal instances in ${TrD}_{k\;}$, and $\left|attack\right|$ is the number of attack instances in ${TrD}_{k\;}$. For example, smart meter 1 receives 20% of the normal class and 12% of the attack class. Algorithm 1 presents the proposed smart meter data-splitting process. Then, each NAN smart meter exchanges its local model parameters to build its data concentrator model by obtaining the average of the parameters. This process is repeated across all data concentrators. Finally, the data concentrators exchange their model parameters with the headend system, which also finds the average to build the global model. The global model tests the final model on the $D_{z\;}\;and\;TeD_{k}$ datasets. After that, the new parameters are exchanged with all smart meters.
**Algorithm 1:** Proposed smart meter data-splitting algorithm.**Data set to split **${TrD}_{k}$**, testing size **z**, client index **i, the total number of clients N**, percentage of normal instances **CN**, percentage of attack instances **CA.**Start****split-SM**$({TrD}_{k}$, z, N)$\mathrm{normal}={TrD}_{k}$[normal]$\mathrm{attack}={TrD}_{k}$[attack]CN_[i] ← generate N random values which sum to 1CA_ [i] ← generate N random values which sum to 1for i to N    normal(i) =normal.sample(|normal|∗CN_i)    attack(i) =attack.sample(|attack|∗CA_i)    normal.drop(normal(i))    attack.drop(attack(i))    Local_data(i) = contact[normal(i),attack(i)]    Local-tra(i), local-val(i) ← split [ Local_data(i), label, z]End for**End**

#### 4.1.2. Data Pre-Processing

This stage includes four main steps: handling missing values, feature preparation, feature scaling, and class balancing.

Real-world data are often incomplete due to corruption or failure when recording. In this work, the mean is used to fill in the missing values [37], calculated as in Equation (11):

(11) $$x¯=\frac{1}{n}\sum_{i=1}^{n}x_{i}$$

where $\bar{x}$ is the mean, n represents the number of rows, and $x_{i}$ gives the value of a row *i*.

For feature preparation, the dataset consists of 79 features some of which are constant or null. Removing these features reduced the dimension of the dataset from 79 to 71 features. Feature scaling aims to ignore the bias at different scales, as features may not contribute equally to the model fitting and learning functions. In this work, a min–max scaler was used to transform all features to within the range 0 and 1 [38], calculated as in Equation (12):

(12) $$x_{i,j}=\frac{x_{i,j}-Min}{Max-Min}$$

where $x_{i,j}$ is the feature, Min is the minimum, and Max is the maximum value.

Last, imbalance usually appears in network flow data as the total number of one class is much larger than the other one. This causes biased models with high non-detection rates. SMOTE is the most popular re-sampling method [39], which balances the data by creating new instances for the minority class. Assuming $X_{i}$ is the nearest value from the k-nearest neighbors (KNNs) of the minority class X, a new synthetic instance $X_{n}$ can be generated as in Equation (13):

(13) $$X_{n}=X+rand\left(0,1\right)*|{X-X}_{i}|,i=1,2\ldots .k$$

where $rand\left(0,1\right)$ represents a random number in the range $\left(0,1\right).$ In particular, this work uses the SMOTE approach for the known attack training set.

#### 4.1.3. Classification Model

CNNs are the most used deep learning models. A CNN architecture comprises four types of layers: convolutional layers, pooling layers, fully connected layers, and an output layer. Convolutional layers are used to find the main features while pooling layers are used to reduce the dimensions of features. Fully connected layers are used to determine the class of the input layer, which, finally, is passed to the output layer [40].

To overcome the overfitting problem, a dropout is used after connecting all features to the fully connected layer.

However, the long short-term memory (LSTM) model was introduced to remedy the issue of vanishing and exploding gradients and has proven to be better suited to the maintenance of long-range connections as a result of its ability to recognize the connections between values at the initial and terminal positions of a sequence. The LSTM architecture is characterized by the relationships between individual cells and three gates: the input gate, the output gate, and the forget gate. The input gate modifies the memory according to the input, while the output gate determines the output according to the input and memory gates [41].

Bidirectional LSTM (biLSTM) consists of two subsurface LSTM layers that process data inputs in forward and backward directions. On a basic level, biLSTM duplicates the primary recursive layer of the neural network and, during training, uses the actual data to construct the primary layer. In contrast, the duplicate layer is a reversed copy of these data. There are two different hidden layers, termed the forward and backward hidden layers. In conjunction, these two layers are merged to generate the output [42].

**Figure 5:**
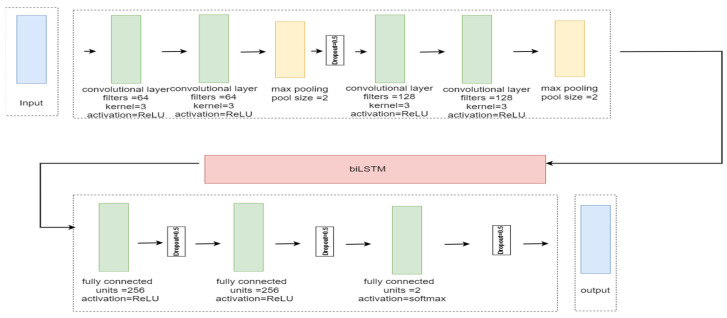
The proposed architecture of the CNN + biLSTM model.

The proposed system employs a hybrid CNN and biLSTM deep learning model, and Figure 5 depicts the proposed architecture. The proposed CNN model consists of four convolutional layers. The number of filters is 64 for the first two convolutional layers and 128 for the latter two. The Rectified Linear Unit (ReLU) activation function is used for each layer, with a kernel size of three. The ReLU is computed as in Equation (14) [43]:

(14) $$ReLU=\left\{\begin{array}{cc} x_{i}, & x_{i}>0\\ 0, & x_{i}\leq 0 \end{array}\right.$$

For each group of two convolutional layers, a pooling layer with a pooling size of two and the max pooling method were used, calculated as in Equation (15) [44]:

(15) $$x_{t}=f_{max\text{-}pooling}\{ReLU(x_{t}*w_{t}+b_{t})\}$$

The first pooling layer for the first group of convolutional layers is followed by a 0.5 dropout rate. The biLSTM layer then follows the second pooling layer for the second group of convolutional layers.

Three fully connected layers come next. A dropout with a 0.5 dropout rate follows each fully connected layer. The first and second layers use the ReLU activation function, whereas the last one uses the SoftMax activation function [45], as described in Equation (16):

(16) $$f_{j}\left(z\right)=\frac{e^{z_{j}}}{\sum_{k=1}^{n}e^{z_{k}}}$$

where *z* is arbitrary with absolute values $z_{j}$, and *n* is the size of the vector.

### 4.2. Proposed Hierarchical Federated Learning-Based Anomaly Detection System

**Figure 6:**
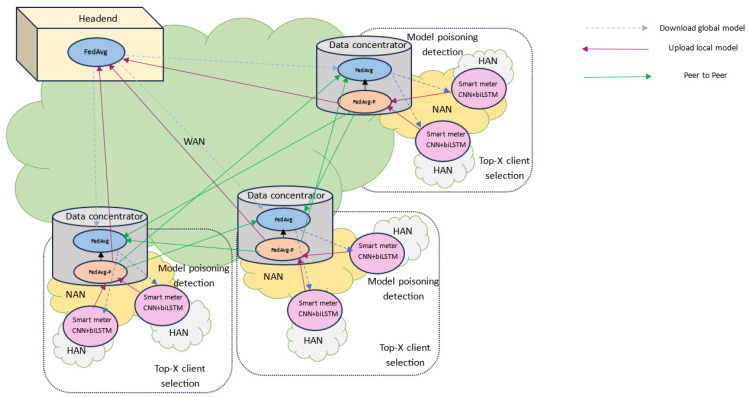
The framework of the proposed hierarchical federated learning-based anomaly detection system.

Figure 6 shows the proposed HFL-ADS for the AMI network. We used two aggregation strategies: FedAvg and our novel performance-based method, FedAvg-P. FedAvg aggregates data concentrator models to obtain the global model, whereas FedAvg-P aggregates smart meter models to obtain the data concentrator model. We also connected all data concentrators via a peer-to-peer connection.

The classical FL strategy of FedAvg is the most frequently employed federated learning aggregation strategy. FedAvg combines local training and global aggregation techniques through averaging model parameters and collects model updates from clients iteratively, typically selecting clients randomly at each iteration [46]. Algorithm 2 presents the FedAvg algorithm.
**Algorithm 2:** Proposed FedAvg algorithm.**Data concentrator model **$W_{D_{j}}^{\left(t+1/2\right)}$, node index k, number of iterations t**, global model **$\omega^{\left(t+1\right)},$** meter dataset **SDi** of size **|SDi|**, whole dataset **SD **of size **|SD|**Start****FedAVG**$(W_{D_{j}}^{\left(t+1/2\right)},\;$k)$\omega^{\left(t+1\right)}=\sum_{j=1}^{k}\left(\frac{\sum_{{i\in D}_{j}}\left|{SD}_{i}\right|}{\left|SD\right|}\;W_{D_{j}}^{\left(t+1/2\right)}\right)$**Return **$\omega_{t+1}$**End**

However, the proposed FedAvg-P selects the top-X clients based on their F1 score. It validates each weight using a predefined global testing set in each data concentrator, and then bans and deletes smart meters that reach a predefined threshold. After that, it aggregates the accepted models to minimize the number of models that require transmission to the headend. Algorithm 3 presents the FedAvg-P algorithm.
**Algorithm 3:** Proposed FedAvg-P algorithm.**Meter index **k, fraction of clients participating C, received global model at iteration t $\omega_{i}^{\left(t\right)},$** meter dataset **SDi** of size **|SDi|**, whole dataset **SD **of size **|SD|**, number of epochs **E, minibatch size B**, learning rate **$\;\eta ,\;\mathrm{smart}\;\mathrm{meter}\;\mathrm{model}\;\omega_{i}^{\left(t+1/2\right)}$**, data concentrator model **$\omega_{D_{j}}^{\left(t+1/2\right)}$**Start****FedAVG-P**$(\omega_{i}^{\left(t\right)}$,k)m ← Max(C. k,1)S_t_ ←$\;(\mathrm{top}\text{-}X\;\mathrm{set}\;\mathrm{of}\;\mathrm{CS}\text{-}P(\omega_{i}^{\left(t\right)},\;$m) clients)For each i ∈ S_t_$\;\;\;\;\;\;\mathrm{If}\;f_{i}\left(\omega_{i}^{\left(t+1/2\right)}\right)\;$> τ         Ban client     Else$\;\;\;\;\;\;\;\;\;\;\omega_{D_{j}}^{\left(t+1/2\right)}=\sum_{i\in D_{j}}^{St}\left(\frac{\left|{SD}_{i}\right|}{\sum_{{i\in D}_{j}}\left|{SD}_{i}\right|}\;\omega_{i}^{\left(t+1/2\right)}\right)$     End ifEnd for**Return**               $\omega_{D_{j}}^{\left(t+1/2\right)}$**CS-P**($\omega_{i}^{\left(t\right)},$m)For each k ∈ m$\;\;\;\;\;\omega_{t+1}^{k}$ ←$\;\mathrm{ClientUpdate}(k,\;\omega_{t}$)    CS ←$\omega_{t+1}^{k}$End for**Return** CS**ClientUpdate**(k,ω)β ← spilt SDi into batches of size BFor i ← E    For b ←β           Train local model ω            $f_{i}\left(\omega_{i}^{\left(t\right)}\right)$ = $\frac{1}{\left|{SD}_{i}\right|}\sum_{(x_{n},y_{n})\in \;{SD}_{i}}ɭ\left(\omega ;(x_{n},y_{n})\right)$           $\omega_{i}^{\left(t+1/2\right)}=\omega_{i}^{\left(t\right)}-\eta \nabla f_{i}\left(\omega_{i}^{\left(t\right)}\right)$    End forEnd For**End**

**Figure 7:**
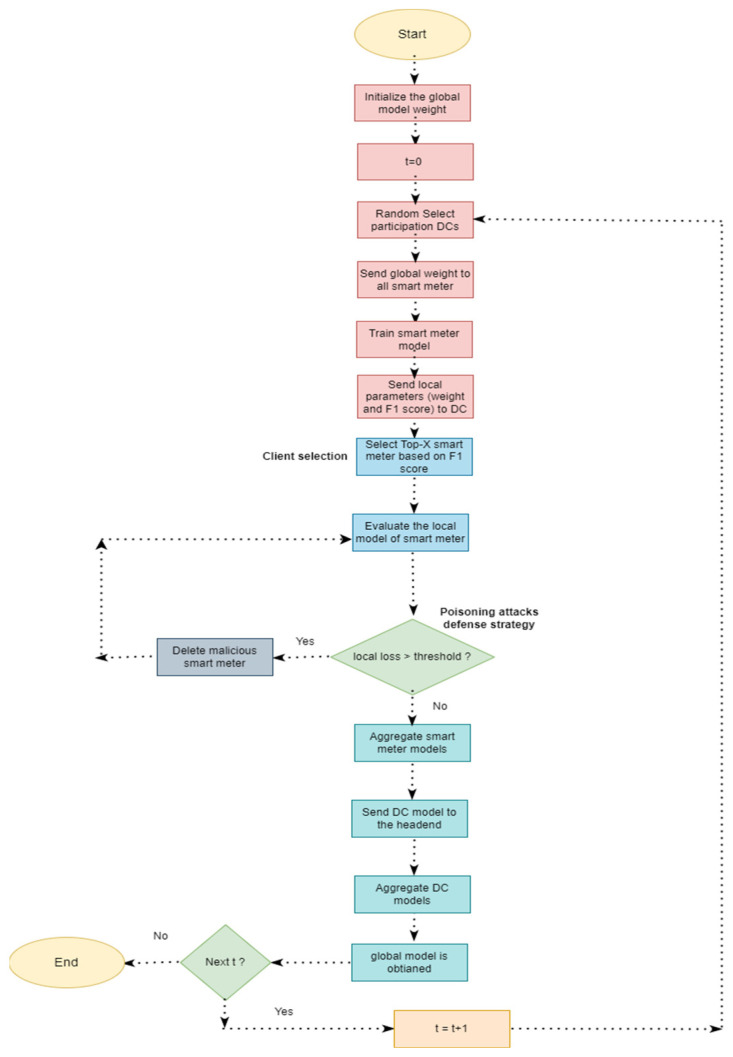
The main process of the proposed framework.

The main process of the proposed system, shown in Figure 7, can be summarized as follows:

Step 1: The headend uploads the global model and initializes the global parameters $\omega_{i}^{\left(t\right)}$;
Step 2: The headend system randomly selects the participant data concentrators and broadcasts the global parameters $\omega_{i}^{\left(t\right)}$;
Step 3: The selected data concentrator broadcasts the global parameters $\omega_{i}^{\left(t\right)}$ to all associated smart meters;
Step 4: The selected data concentrator uses the proposed FedAvg-P algorithm;
Step 5: In the FedAvg-P algorithm, each smart meter uses its local data to train its CNN+biLSTM model;
Step 6: After the smart meter’s local model is trained, it sends its local model parameters, local data size, and F1 score to the data concentrator;
Step 7: The data concentrator receives the model parameters of all smart meters, ranks them based on their F1 score, and then selects the top-X smart meters;
Step 8: The data concentrator trains the selected smart meter model to validate and ban malicious smart meters;
Step 9: If the loss of the smart meter model is greater than the threshold, ban and delete it; otherwise, accept the model;
Step 10: The accepted models are aggregated by the data concentrator, and its model is obtained;
Step 11: After obtaining the model, the data concentrator uploads the model parameters to the headend system, where the FedAvg-P aggregation is ended;
Step 12: The obtained data concentrator model is sent to all data concentrators using a peer-to-peer connection;
Step 13: The headend system receives the data concentrator model parameters from the selected data concentrators, aggregates them using FedAvg, and obtains a global model;
Step 14: After obtaining the global model, the headend broadcasts it to all smart meters;
Step 15: The data concentrator receives other data concentrator model parameters, aggregates them using FedAvg, and obtains a global model.

Algorithm 4 depicts the proposed HFL-ADS algorithm.
**Algorithm 4:** Proposed peer-to-peer HFL-ADS algorithm.**Smart meter index **N, data concentrator index M**, fraction of clients participating C, number of rounds** *t***, meter dataset **SDi ** of size **|SDi|**,**$\;\mathrm{global}\;\mathrm{model}\;\omega^{\left(t+1\right)}$**,** received global model at iteration t $\omega_{i}^{\left(t\right)}$**, smart meter model **$\omega_{i}^{\left(t+1/2\right)}$**, data concentrator model **$\omega_{D_{j}}^{\left(t+1/2\right)}$:
**Start**$\mathrm{Initialize}\;\mathrm{the}\;\mathrm{global}\;\mathrm{model}\;\mathrm{and}\;\mathrm{send}\;\mathrm{it}\;\mathrm{to}\;\mathrm{all}\;\mathrm{smart}\;\mathrm{meters}\;\omega_{i}^{\left(t\right)}$.For each round tm ← Max(C. M, 1)S_t_ ← (random set of m data concentrator)For each j ∈ S_t_         $\omega_{D_{j}}^{\left(t+1/2\right)}=$ **FedAVG-P**$(\omega_{i}^{\left(t\right)},\;$N)        $\mathrm{Send}\;W_{D_{j}}^{\left(t+1/2\right)}$ to all S_t_        $\omega^{\left(t+1\right)}$ $=\mathrm{FedAVG}(\;W_{D_{j}}^{\left(t+1/2\right)},\;$S_t_)End For$\omega^{\left(t+1\right)}$ $=\mathrm{FedAVG}(\omega_{D_{j}}^{\left(t+1/2\right)},\;$S_t_)For each j ∈ S_t_        $W_{D_{j}}^{\left(t+1\right)}$ $=\omega^{\left(t+1\right)}$        For i←N        $\omega_{i}^{\left(t+1\right)}$ $=W_{D_{j}}^{\left(t+1\right)}$        End For    End forEnd for**End**

## 5. Performance Evaluation

The experiments were conducted based on five scenarios. First, a comparative experiment was undertaken to compare the proposed deep learning hybrid model (CNN+biLSTM) with CNN, LSTM, and biLSTM. Second, a comparative experiment was carried out in which the proposed FedAvg-P was compared to a random selection strategy. This was followed by simulated poisoning attacks to test the system’s efficiency. Fourth, a thorough simulation of the proposed system and its mechanisms was conducted. Finally, we compared the proposed system with those presented in the related literature.

### 5.1. Performance Comparison of the Proposed Model and State-of-the-Art-Deep Learning Models

**Figure 8:**
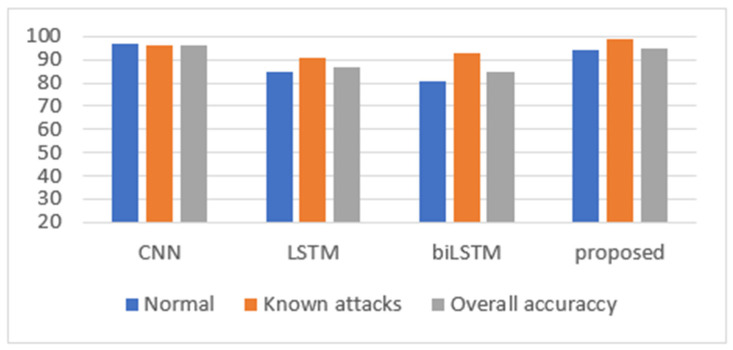
The experimental results for the CICIDS2017 dataset.

First, we evaluated the proposed CNN+biLSTM model by comparing it with CNN, LSTM, and biLSTM for known and zero-day attacks. Table 2 shows an accuracy (%) comparison of the CICIDS2017 dataset for known attacks; Figure 8 graphically represents these results.

In Table 2, for single models, the CNN model performed best in classifying normal flows (97%) and detecting known attacks (96%), when compared with LSTM and biLSTM. Moreover, the CNN model well classified normal flows (97%), with a slight difference in detecting known attacks (96%) and a noticeable difference when compared with other single deep learning models. In addition, all single models showed a high performance in detecting attacks. On the other hand, LSTM and biLSTM had lower accuracy in classifying normal conditions than when detecting known attacks. Moreover, the CNN model reached a high overall accuracy of about 96.32%.

Table 3 presents the accuracy comparison of the CICIDS2017 dataset for zero-day attacks. The results indicated that CNN achieved the lowest result (60%), followed by LSTM (63%), while biLSTM achieved the highest result (66%). The CNN defined most flows as normal, whereas LSTM and biLSTM classified them as attacks. Generally, biLSTM showed the best result when defining zero-day attacks.

Accordingly, from Table 2 and Table 3, the results indicated that the CNN provided the best result in terms of detecting known attacks, while biLSTM performed the best in detecting zero-day attacks. In this work, a hybrid model based on CNN and biLSTM was developed. From Table 2, it can be seen that CNN+biLSTM achieved the highest result in detecting known attacks (at about 99%), while the average result was between those for CNN and biLSTM when classifying normal and overall accuracy (about 94% and 95.46%, respectively). For the zero-day attack in Table 3, the proposed CNN+biLSTM model also obtained the highest value (about 70%). This means that combining CNN and biLSTM in a single hybrid model increased the model’s ability to detect and isolate known and zero-day attacks.

**Figure 9:**
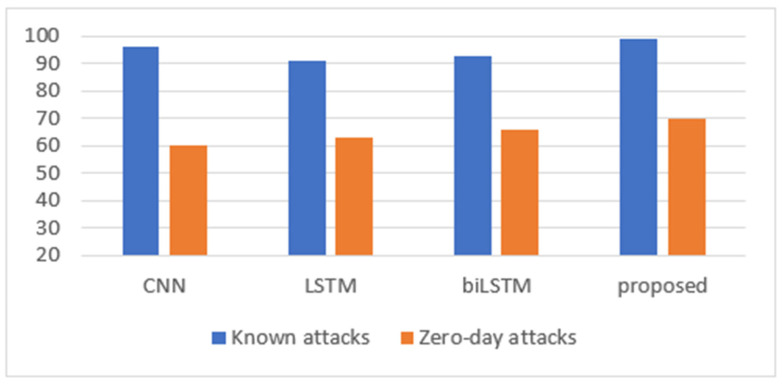
Detection rate for known and zero-day attacks for the CIC-IDS2017 dataset.

Additionally, Table 4 represents the DR of the models, while Figure 9 presents these results graphically. They show that the CNN+biLSTM system obtained the highest results for known and zero-day attacks: about 99% and 70%, respectively.

**Figure 10:**
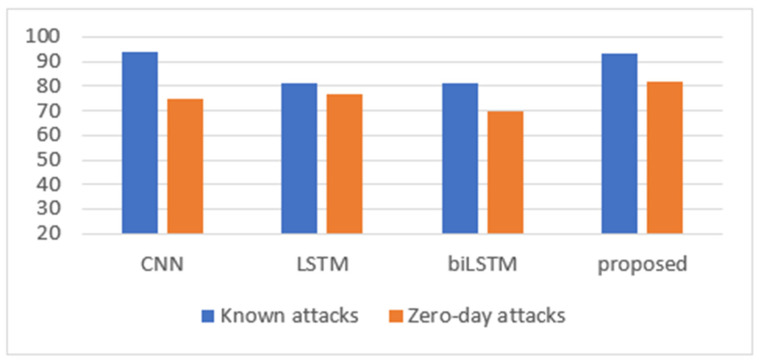
F1 score for known and zero-day attacks for the CIC-IDS2017 dataset.

Moreover, Table 5 presents the F1 scores of the models, and Figure 10 depicts these results graphically. For known attacks, the CNN model reached the highest value (94%), with a slight difference compared to the CNN+biLSTM model (93%). On the other hand, CNN+biLSTM obtained the highest value for detecting zero-day attacks (82%).

**Figure 11:**
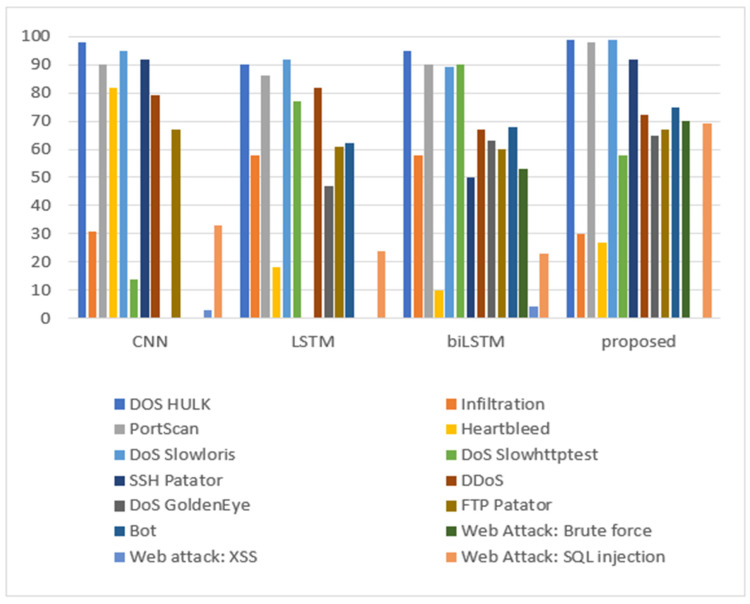
Detection accuracy per class for both known and zero-day attacks using the CIC-IDS2017 dataset.

Accordingly, Table 6 and Figure 11, define the detection accuracy per attack class for all models. For known attacks, the SSH Patator was detected by models containing CNN or biLSTM only. The proposed model was highly accurate in detecting DoS Hulk, DoS Slowloris, and PortScan attacks. Concerning the rest of the known attacks, the detection accuracy varied between the models; overall, the proposed CNN+biLSTM model obtained average results compared to CNN and biLSTM.

However, for the zero-day attack, combining CNN and biLSTM in a single hybrid model enhances the detection accuracy for DoS GoldenEye, FTP Patator, Web Attack: Brute Force, Bot, and Web Attack: SQL Injection. Moreover, Web attack: XSS has shown poor performance for all models and 0 accuracy for the proposed model. For the DDoS, the proposed CNN+biLSTM model achieved the average results of CNN and biLSTM.

### 5.2. Performance of the Proposed System with Random Selection

The next experiment studied the efficiency of the proposed client selection method in comparison to the proposed system with random selection. Table 7 presents the performance of the proposed system with a random selection strategy.

**Figure 12:**
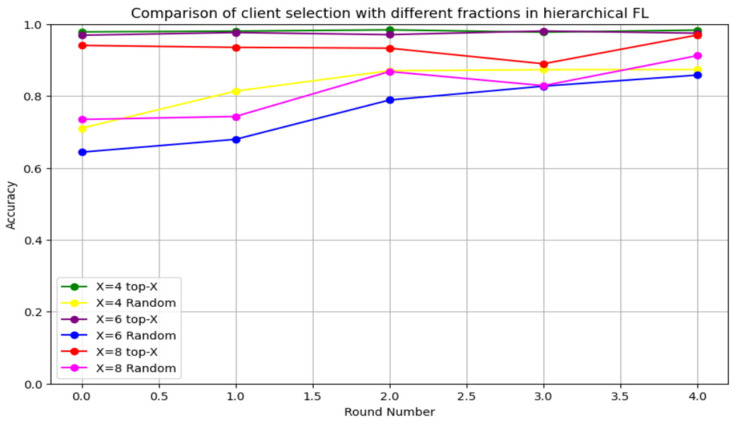
Accuracy evaluation of different X sizes using the CICIDS2017 dataset.

We initially investigated the impact of varying X on HFL performance. We used three different X sizes, X = 0.4, X = 0.6, and X = 0.8, with 10 clients. A performance evaluation with different X values using the CICIDS2017 dataset over a total of five rounds is illustrated in Figure 12. The proposed client selection method—top-X based on performance—significantly improved the accuracy compared to random selection across all X values. However, the effect of X was considerable, particularly for smaller X values. X = 0.8 yielded the lowest accuracy performance, while X = 0.2 generated the highest accuracy enhancement. Choosing the top-X means allowing only the X-highest clients to participate in aggregation and preventing the low-performance clients from affecting the global model performance. Additionally, decreasing the value of X leads to an increase in the global model’s performance. Thus, the CICIDS 2017 dataset evaluation demonstrated that FedAvg-P can improve the performance of FedAvg.

**Figure 13:**
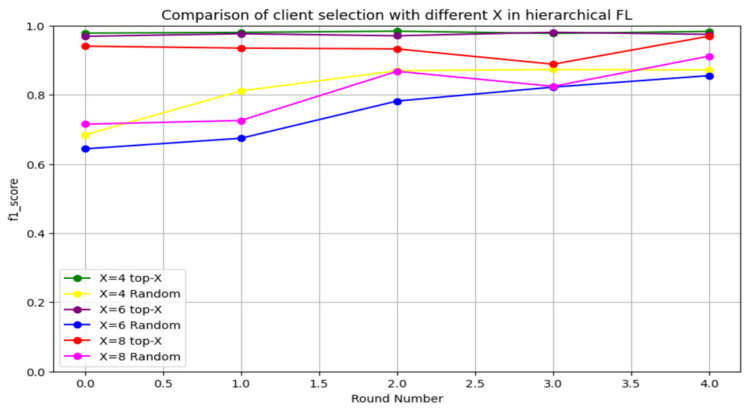
F1 score evaluation of different X sizes using the CICIDS2017 dataset.

Furthermore, we investigated the F1 score to provide a comprehensive evaluation, as illustrated in Figure 13. The performance of the proposed FedAvg-P was superior to random client selection in FedAvg. The proposed FedAvg-P enhanced the overall performance in terms of accuracy and F1 score.

### 5.3. Performance of the Proposed System When Simulating Poisoning Attacks

The following experiment studied the efficiency of the proposed poisoning attack defense method through simulated attacks on two different smart meters in two separate NANs. The comparison involves the performance results with and without the proposed FedAvg-P, as shown in Table 8.

**Figure 14:**
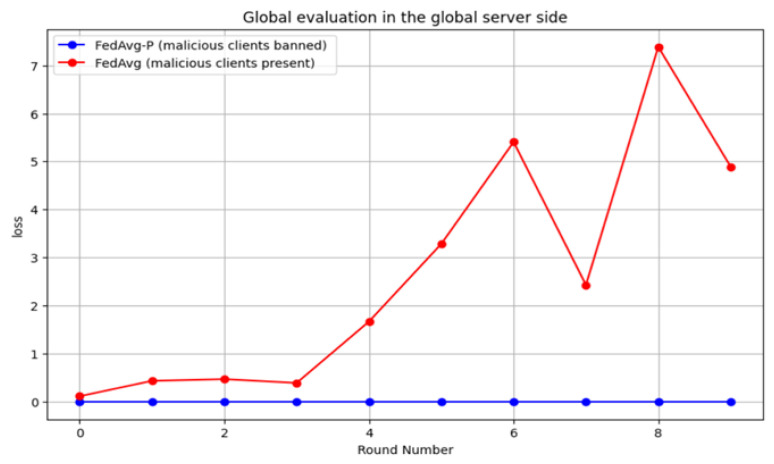
The loss rate of the system with and without detecting poisoning attacks as the number of rounds increases.

The results show that the undetected model poisoning attacks in FedAvg in the HFL system led to a degraded global model performance in terms of accuracy, DR, and F1 score. Moreover, the target of the poisoning attacks was achieved, minimizing the global accuracy to about 49%. On the other hand, the proposed FedAvg-P could isolate malicious smart meters and ban them from the FL system without affecting the global model performance, reaching 89% accuracy. Figure 14 compares the loss rate of the system with and without detecting poisoning attacks as the number of rounds increases. It shows that detecting attacks keeps the loss rate low (near 0), which is the goal of the FL system.

**Figure 15:**
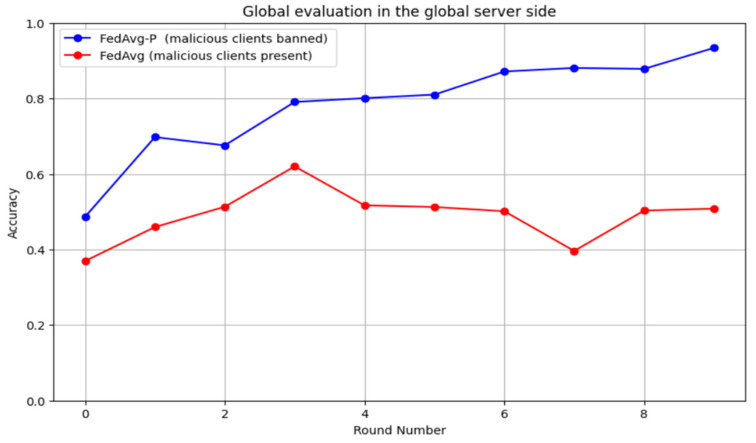
The accuracy rate of the system with and without detecting poisoning attacks as the number of rounds increases.

**Figure 16:**
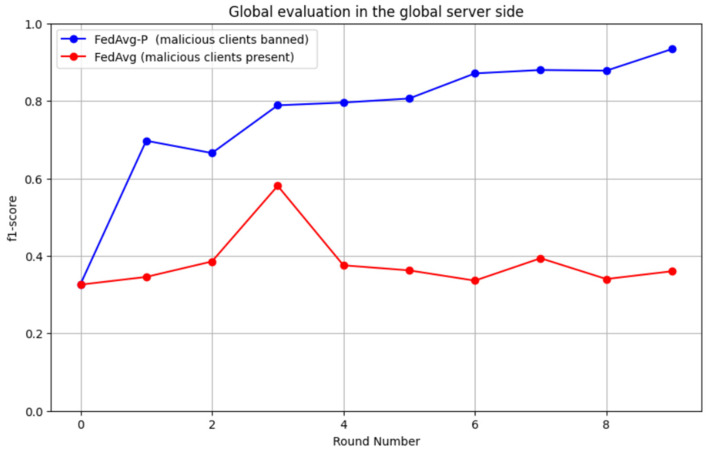
F1 score rate of the system with and without detecting poisoning attacks as the number of rounds increases.

In contrast, malicious smart meters led to huge increases in the loss rate each round. Furthermore, Figure 15 compares the system’s accuracy with and without detecting poisoning attacks as the number of rounds increases. It shows that poisoning attacks target the model’s accuracy by minimizing it in each round. The proposed detection method removes the effect of the attacks and shows clear enhancement in each training round. Finally, Figure 16 illustrates the relationship between the F1 score and the number of rounds for the system with and without the detection of poisoning attacks. Poisoning attacks also led to the deterioration of F1 scores; however, the proposed system maintained high F1 score values in every round.

### 5.4. Comprehensive Simulation of the Proposed System with the Proposed Mechanisms

The next experiment was designed to assess the efficiency of the proposed deep learning CNN-biLSTM model based on HFL to detect known and zero-day attacks in the CICIDS 2017 dataset. The system included two data concentrators and five smart meters for each data concentrator and was trained over a total of five rounds. Moreover, the system selected the top-X smart meters for aggregating the data concentrator model, where X = 2. The system included model poisoning attacks on two different smart meters in two NANs. Table 9 details the performance of the proposed system.

**Figure 17:**
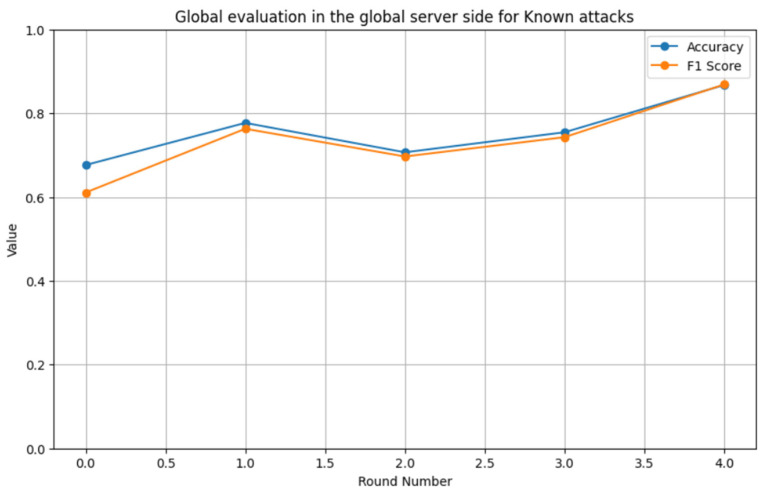
Relationship between the accuracy, F1 score, and number of rounds for known attacks.

**Figure 18:**
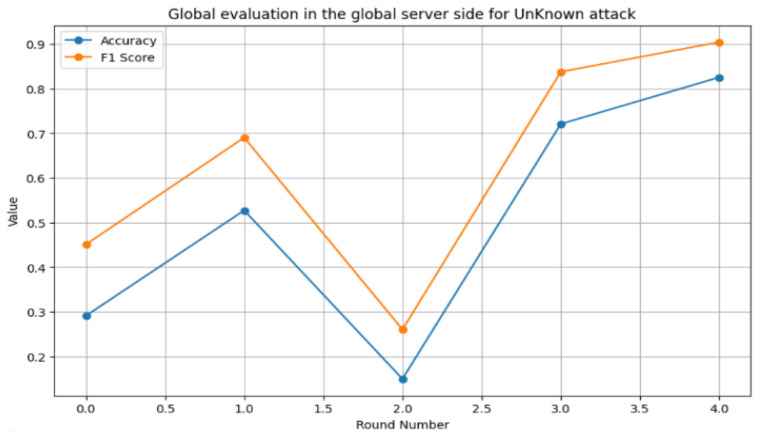
Relationship between the accuracy, F1 score, and number of rounds for zero-day attacks.

The results demonstrate that the proposed system can effectively develop a reliable anomaly detection system for AMI networks. The system revealed its ability to detect known attacks with an accuracy of 86% and an F1 score of 86% for the CIC-IDS2017 dataset, while achieving a high F1 score (90%) and accuracy (82%) for zero-day attacks in the same dataset. In addition, the system selected the high-contribution smart meters based on their local parameters due to their effect on the heterogeneous environment of the AMI network. Furthermore, these results indicate its ability to detect and remove malicious smart meters from the system. Figure 17 illustrates the relationship between accuracy, F1 score, and the number of rounds for known attacks, while Figure 18 shows the relationship between accuracy, F1 score, and the number of rounds for zero-day attacks. These figures show that increasing the number of rounds improves system performance for both known and zero-day attacks.

### 5.5. Performance Comparison of the Proposed Model and Related Works

Finally, we evaluated the proposed system by comparing it with other methods described in the Related Works section. The result was compared to other methods in Table 1. While all these methods rely on federated learning to detect known attacks, they each used distinct datasets, and we found no study that used the same dataset with federated learning.

## 6. Conclusions and Future Works

This work proposes a performance-based HFL-ADS to detect known and zero-day attacks using a DL approach. The proposed DL model is based on CNN + biLSTM and includes the following stages: data collection, data pre-processing, and model classification. Within the HFL system, the participants collaboratively learn the global model by exchanging their local parameters without compromising data privacy. The data concentrator in the proposed system uses two aggregation strategies: First, it uses a novel aggregation strategy called FedAvg-P, which selects the participating smart meters based on their local model performance, and then authenticates each smart meter to ban model poisoning attacks and obtain its local model. Next, the data concentrator aggregates its model parameter with other data concentrator model parameters using the classical FedAvg via a peer-to-peer connection. The headend system then uses only FedAvg to aggregate the data concentrator models to obtain the global model. The results indicate the suitability of the proposed system for this task, as it protected against most zero-day attacks present in the CIC-IDS2017 dataset when compared with state-of-the-art deep learning models (CNN, LSTM, and biLSTM) in a heterogeneous environment. Additionally, the proposed system achieved the highest values compared to the random selection strategy and maintained high performance under poisoning attacks. Therefore, the proposed system has the potential to be used in the development of a secure and reliable ADS for AMI networks. Future research could build upon these findings by adding privacy preservation techniques. Furthermore, client selection at the data concentrator level to improve global performance could also be investigated.

## Figures and Tables

**Table 1:** Summary of the literature on FL detection systems.

Ref.	Year	Performance Enhancing Approach	Environment	Aggregation Strategy	Dataset	FL/HFL	Evaluation Metrics (%)
[25]	2022	Peer-to-peer/Privacy preservation	IoT	FedAvg	NF-BoT-IoT-v2	HFL	DoSAccu = 95 TheftAccu = 63
[26]	2022	-	AMI	FedAvg	NSL-KDD	HFL	Accu, recall, and F1 = 99
[27]	2018	-	SDN	Blockchain	DARPA		ER= 12, DR =99.42
[28]	2023	-	IIoT	Flow aggregator	Edge-IIoTset	FL	Accu = 92
[29]	2022	Privacy preservation	-	FLTrELM	NSL-KDD, KDD99, and ISCX2012	FL	Accu = 70–99
[30]	2023	Peer-to-peer/criteria-based aggregation method	CIoMT	FedAvg	COVID-19 patients	FL	Accu = 90
[31]	2023	CS-based local parameter	IIoT	FedAcc	MNIST and F-MNIST	FL	MNISTAccu = 82 F-MNISTAccu = 56
[32]	2023	CS-based local parameter	-	FedChoice	CIFAR-10 and other	FL	Accu = 86.4

**Table 2:** Accuracy (%) comparison for the CIC-IDS dataset (known attacks).

Model	Normal	Known	Overall
CNN	97	96	96.32
LSTM	85	91	86.98
biLSTM	81	93	84.80
CNN+biLSTM	94	99	95.46

**Table 3:** Accuracy (%) comparison for the CIC-IDS dataset (zero-day attacks).

Model	Zero-Day
CNN	60
LSTM	63
biLSTM	66
CNN+biLSTM	70

**Table 4:** Detection rate (%) comparison for the CIC-IDS dataset.

Model	Known	Zero-Day
CNN	96	60
LSTM	91	63
biLSTM	93	66
CNN+biLSTM	99	70

**Table 5:** F1 score (%) comparison for the CIC-IDS dataset.

Model	Known	Zero-Day
CNN	94	75
LSTM	81	77
biLSTM	79	80
CNN+biLSTM	93	82

**Table 6:** The detection accuracy per attack class for all models.

		CNN	LSTM	biLSTM	CNN+biLSTM
Known Attacks	DoS Hulk	98	90	95	99
	Infiltration	31	58	58	33
	PortScan	90	86	90	98
	Heartbleed	82	18	10	27
	DoS Slowloris	95	92	89	99
	DoS Slowhttptest	14	77	90	58
	SSH Patator	92	0	50	92
Zero-day Attacks	DDoS	79	82	67	72
	DoS GoldenEye	0	47	63	65
	FTP Patator	67	61	60	67
	Bot	0	62	68	75
	Web Attack: Brute force	0	0	53	70
	Web attack: XSS	3	0	4	0
	Web Attack: SQL injection	33	24	23	69

**Table 7:** Performance (%) of the proposed system with a random selection strategy.

Model	X	Accuracy	F1 Score
FedAvg	4	87	87
FedAvg-P		98	98
FedAvg	6	85	85
FedAvg-P		97	97
FedAvg	8	91	91
FedAvg-P		96	96

**Table 8:** Performance (%) of the proposed system under poisoning attack.

Model	Accuracy	DR	F1 Score
FedAvg	49	49	34
FedAvg-P	89	89	89

**Table 9:** Performance (%) of the proposed system.

Known Attack		Zero-Day Attack	
Accuracy	F1	Accuracy	F1
86	86	82	90

## Data Availability

Part of the data are available upon request.
